# The ISIS-TTRRx-CS2 phase 3 study in patients with familial amyloid polyneuropathy: Baseline results of the first 100 patients for the NIS, NIS+7 and mNIS+7 using different methods of scoring: identification of consistencies and key differences

**DOI:** 10.1186/1750-1172-10-S1-O22

**Published:** 2015-11-02

**Authors:** Rito Bergemann, Elizabeth Ackermann, Brett Monia, Andrew Shenker, Peter Dyck

**Affiliations:** 1 GSK, VEO Rare Diseases, Brentford, TW8 9GS, UK; 2 Isis Pharmaceuticals, CD, Carlsbad, CA 92010, USA; 3 GSK, Rare Disease Unit, King of Prussia, PA 19406, USA; 4 Mayo Clinic, Department of Neurology, Rochester, MN 55905, USA

## Background

Familial amyloid polyneuropathy (FAP) is a devastating disease with continuous progression over time leading to complete disability, bedridden status, and ultimately death within 10 to 15 years from symptom onset. In order to document disease progression, it is necessary to have valid instruments which allow assessment of the severity of the sensory, motor and autonomic neuropathy components of the disease. Over the last decades, multiple related instruments have been used and modifications developed to measure disease progression in FAP patients in clinical trial settings. These include the Neuropathy Impairment Score (NIS), originally called Neuropathy Disability Score, the Neuropathy Impairment Score for the Lower Limbs (NIS-LL), the Neuropathy Impairment Score +7 (NIS+7) and the modified NIS +7 (mNIS+7). The NIS scores were first used to quantitate neuropathic impairment in a series of therapeutic trials in chronic inflammatory demyelinating polyradiculoneuropathy and were also used as primary endpoints in clinical trials for FAP in the completed diflunisal (NIS+7) and tafamidis (NIS-LL) Phase 3 trials. Building on experience from these trials, modifications to the NIS+7 were developed (Suanprasert et al, 2014, J Neurol Sci) and are currently being applied in the ongoing ISIS-TTRRx-CS2 (mNIS+7 Isis) and patisiran (mNIS+7 ALN) Phase 3 trials. Even though the mNIS+7 Isis and mNIS+7 ALN scores are very similar, they are not identical. The details in the assessment and scoring for the various versions of the NIS based scores are described and their scoring methods applied to baseline data collected on the first 100 patients in the ISIS-TTRRx-CS2 trial to better understand and illustrate the key differences.

## Methods

The demographics and neuropathic status of the first 100 patients enrolled in the ISIS-TTRRx-CS2 trial were analysed. At baseline, all assessments needed to calculate NIS, NIS-LL, NIS+7 and mNIS+7 Isis were obtained in duplicate. All assessments required to calculate the mNIS+7 ALN were also obtained except for postural blood pressure, which accounts for < 1% of the mNIS+7 ALN score. Data are presented using descriptive statistics.

## Results

The most common mutation was Val30Met followed by Thr60Ala. The mean age was 62 yrs (range 27-81) and 76% were male. The differences in the NIS based scales and impact of the changes in relative weights of the sub-scores were investigated. The results will be presented for each score and scoring method and will include the baseline data for NIS (median 41; range 3 - 106), NIS+7 (median 57; range 10 – 120) and mNIS+7 Isis (median 74; range 10 - 163).

## Conclusion

As interest in developing improved therapeutics for FAP continues to grow and as more physicians are recognizing and following FAP patients in clinical practice, it is important that the differences between the various instruments used to measure disease progression are acknowledged, compared and understood.

